# Comprehensive analysis of epigenetics mechanisms in osteoporosis

**DOI:** 10.3389/fgene.2023.1153585

**Published:** 2023-03-28

**Authors:** Yuzhu Chen, Yumiao Sun, Xiangyu Xue, Huanzhi Ma

**Affiliations:** ^1^ The Second Affiliated Hospital of Harbin Medical University, Harbin, Heilongjiang, China; ^2^ Harbin Medical University, Harbin, Heilongjiang, China; ^3^ Department of Orthopedics, Shandong Provincial Hospital Affiliated to Shandong First Medical University, Jinan, China

**Keywords:** epigenetics, osteoporosis, DNA methylation, histone modification, non-coding RNA (ncRNAs)

## Abstract

Epigenetic modification pertains to the alteration of genetic-expression, which could be transferred to the next generations, without any alteration in the fundamental DNA sequence. Epigenetic modification could include various processes such as DNA methylation, histone alteration, non-coding RNAs (ncRNAs), and chromatin adjustment are among its primary operations. Osteoporosis is a metabolic disorder that bones become more fragile due to the decrease in mineral density, which could result in a higher risk of fracturing. Recently, as the investigation of the causal pathology of osteoporosis has been progressed, remarkable improvement has been made in epigenetic research. Recent literatures have illustrated that epigenetics is estimated to be one of the most contributing factors to the emergence and progression of osteoporosis. This dissertation primarily focuses on indicating the research progresses of epigenetic mechanisms and also the regulation of bone metabolism and the pathogenesis of osteoporosis in light of the significance of epigenetic mechanisms. In addition, it aims to provide new intelligence for the treatment of diseases related to bone metabolism.

## 1 Introduction

Bone is composed of four distinct cell types: osteoprogenitor cells, osteoblasts (OBs), osteocytes, and osteoclasts (OCs). Mesenchymal stem cells derived from bone marrow possess the ability to become OBs, thus permitting the development of fresh bone. OCs are distinct from OBs in that they dissolve bone tissue by expelling mononuclear macrophages from bone marrow. OBs and OCs help to maintain the correct amount of minerals in bones and cartilage, as well as the strength of the bones, during their development and renewal (Chen et al., 2018).

The interaction, differentiation, and associated roles of OBs of the mesenchymal type and OBs of the hematopoietic type are crucial for bone production and resorption in the adult skeleton. Differing perspectives, activation, and induction of apoptosis in OBs and OCs are tightly regulated to balance bone turnover. Once the regulatory process becomes abnormal, it often leads to an imbalance in bone homeostasis. Fragility fractures represent the most significant risk for osteoporosis, the leading cause of age-related disability and death. If the rate of bone formation is not equal to the rate of bone loss, it can cause the onset of osteoporosis. Due to osteoporosis, bone strength and structure are weakened, raising the likelihood of fracturing (Siris et al., 2014). According to publicly available data, China has the highest number of people who have osteoporosis. By 2022, there will be more than 90 million people with osteoporosis in China, representing approximately 7% of the country’s total population. Related studies have demonstrated a link between a genetic variation, alterations in DNA methylation, and the likelihood of developing osteoporosis or a fracture due to surgery in humans. There are now genome-wide association studies (GWAS) demonstrating genetic variation and DNA methylation alterations in the development of human osteoporosis and surgical fractures (Yang et al., 2020). In addition, Kemp et al.'s Genome-Wide Association Study uncovered numerous loci connected to Bone Mineral Density, however, these loci only accounted for a relatively low amount of the overall variance in the bone phenotype (∼12%) (Kemp et al., 2017).

Osteoporosis is generally accepted as a genetically influenced multifactorial disease. Epigenetics is the branch of genetics that studies the inheritance of expression of genes without DNA sequences. It is considered the “new genetics” and is currently at the forefront of life sciences. The expression of genes can be altered through post-transcriptional processes like DNA methylation, histone modifications, and ncRNAs regulation, which are all known collectively as epigenetics. Of these, epigenetic mechanisms involving DNA methylation have been studied most extensively. Epigenetic factors strongly influence OB or OC differentiation and activity and are related to the development of osteoporosis (Marini et al., 2016; van Meurs et al., 2019). Recently, it has been shown that epigenetics has an important role in regulating the differentiation of bone marrow mesenchymal stem cells (MSCs) into OBs and OCs by regulating the expression of genes in the estrogen endocrine pathway, the Wnt/β-catenin signaling pathway, the OPG/RANKL/RANK system, the vitamin D endocrine pathway and other metabolic-related pathways, and thus regulating the differentiation and activity of OBs and OCs, thus participating in the development of osteoporosis (Park-Min, 2017).

Osteoporosis results from an interaction between genetics, epigenetics, and the environment. Osteoporosis is largely determined by epigenetic factors rather than genetic factors. The alteration of this element has a considerable impact on the interaction between various pathways and the proteins that control the formation of bone cells. While most previous studies have been reviewed based on one point of epigenetics, this article delves into the triple influence of epigenetics on the progression of osteoporosis, which includes DNA methylation, histone modifications and ncRNAs, and is more comprehensive and specific compared to most previous articles. It examines the research surrounding osteoporosis, discusses possible underlying mechanisms from multiple perspectives, and suggests epigenetic preventive measures that provide new perspectives for clinical research.

## 2 The roles of DNA methylation modification in the occurrence of osteoporosis

DNA methylation, which is the incorporation of a methyl moiety to the fifth atom of a cytosine molecule on a CpG island, can suppress gene expression, but this effect can be reversed. An increasing number of studies demonstrate the epigenetic effects of environmental factors, including behavior, nutrition, chemicals, and industrial pollutants. Environmental influences throughout a person’s life are believed to have effects on their health, with epigenetic changes like DNA methylation beginning in the womb (Tiffon, 2018). DNA methylation is also closely associated with the development of osteoporosis. An inquiry into the DNA methylation patterns in femoral trabecular tissues from individuals suffering from osteoporotic hip fractures demonstrated a negative relationship between methylation and other variables and the totality of all genetic expression (23,367 CpG sites from a total of 13,463 genes were examined for methylation) (Delgado-Calle et al., 2013). Numerous essential genes, proteins and pathways involved in bone-related cellular activities, including sclerostin (SOST), estrogen receptor alpha (Erα), DNA methyltransferase (DMNT), hedgehog interacting protein (HHIP), runt-related transcription factor 2 (Runx2), nuclear factor erythroid 2- related factor 2 (Nrf2), receptor activator of nuclear factor-kappa B (RANK), nuclear factor-kB receptor activator ligand (RANKL), osteoprotegerin (OPG), wnt/β-catenin signaling pathway, and notch signaling pathway are co-regulated by DNA methylation status. The specific contribution of each gene, protein, or expression pathway to osteoporosis is detailed below.

### 2.1 SOST

SOST, an osteosclerotic protein, is an osteocyte-derived component that has multiple functions in various tissues. As OBs and OCs grow and expand, the SOST protein which is present in high amounts hampers the ripening of OBs and fosters the demise of OBs on purpose.

SOST protein is an important component in the Wnt/β-catenin signaling pathway, as it is vital for the governing of bone and glucose metabolism. Furthermore, it has a strong bond with its co-receptor LRP5/6, which has the capability to impede the development of skeletal structure by obstructing the Wnt/β signaling pathway activation (Gaudio et al., 2014; You et al., 2015). Patients without the SOST gene presented a higher bone density, a trait that is normally associated with the secretion of SOST protein by OBs (van Lierop et al., 2013).

Osteoporosis epigenetic studies in the early days generally concentrated on a limited number of genetic elements that have fundamental functions in bone formation. An example of one research study compared the degrees of DNA methylation in the vicinity of the promoter region the SOST gene from skeletal tissue specimens for examination obtained from four women who have gone through menopause and have been diagnosed with osteoporosis and four healthy individuals (Reppe et al., 2015). This research revealed a notable augmentation in the SOST promoter’s methylation levels among individuals afflicted with osteoporosis. In a distinct group of 63 postmenopausal women, including 27 individuals with the condition and 36 unaffected people, it displayed this. A study data revealed a direct correlation between the concentration of SOST mRNA in bone specimens, the level of sclerostin (a factor that inhibits bone development) in the blood, and the thickness of the bones. However, a 2019 study reported conflicting results comparing femur tissues from 16 Chinese osteoporosis patients and 16 controls, showing that SOST gene expression levels were significantly increased (mRNA and protein), and SOST-activated children showed mild hypomethylation (Cao et al., 2019). More studies are required to acquire a greater comprehension of the association between SOST methylation changes and osteoporosis.

### 2.2 Erα

Many women who have gone through menopause suffer from postmenopausal osteoporosis. After menopause, a type of osteoporosis called postmenopausal osteoporosis can occur, caused by the decrease in the hormone estrogen which leads to an imbalance between the destruction and creation of bones. The Erα gene is situated on 6q25.1 of chromosome 6 and is composed of 140,000 nucleotide base pairs. Estrogen acts on bone through ER and affects bone reconstruction. The Erα gene can affect bone by regulating the amount of estrogen produced. Therefore, estrogen deficiency predisposes to osteoporosis. Estrogen binds to Erα, which then sets off a series of ERα-associated pathways. Changes in SOST promoter methylation have been observed to influence estrogen’s connection to Erα (Shan et al., 2019). In addition, estrogen can enhance Erα gene expression through DNA methylation, which also affects the biological function of estrogen to some extent. It is proposed that the Erα gene is a critical regulator in postmenopausal osteoporosis and could be implicated in the cause of osteoporosis (Kondo et al., 2014). Exploring the influence of Erα gene alterations on the progression of postmenopausal osteoporosis could be beneficial.

### 2.3 DNMT

DNMT facilitates the incorporation of methyl molecules onto CpG islands, which weakens the integrity of the genome and hinders the manifestation of genes (Okano et al., 1999). DNMT1 keeps the methylation pattern of DNA which has been partially methylated, while DNMT3A and DNMT3B are more active on DNA which has not been methylated or only partially methylated.

The animal model of homocysteinemia with osteoporosis resulted in a higher expression of DNMT1, which then triggered an upsurge in methylation of the OPG promoter, ultimately resulting in reduced OPG transcription and an increase in RANKL expression, thus allowing OC formation to take place and resulting in a decrease in bone density (Behera et al., 2018).

Nishikawa et al. revealed that DNMT3A is able to facilitate DNA methylation by using S-adenosylmethionine (SAM) and also inhibit interferon regulatory factor 8 (IRF8) to govern OC production, a pivotal inhibitor of the OC phenotype which has to be epigenetically deactivated to allow OC production. In DNMT3A knockout mice, OC precursors do not differentiate and mature effectively *in vitro*. The DNMT3A knockout mouse had fewer activated OCs and more bone mass than the normal control group (Zhao et al., 2009). When the menopausal model of ovariectomized women were given the DNMT3A inhibitor theaflavin 3,30-diacid (TF-3), the quantity of active OCs diminished while the amount of bone mass grew. It is recommended to prevent osteoporosis. Suppressing DNMT3A activity may be an efficient way to avert osteoporosis and treat postmenopausal surgery (Nishikawa et al., 2015).

### 2.4 HHIP

Hedgehog (Hh) signaling pathways are a group of pathogenetic signals that regulate embryogenesis, organogenesis, adult stem cell homeostasis, and tissue maintenance and are involved in various tumors. By attaching Hh to SMO in this signal, PTCH1’s obstruction is lessened and the quantity of Gli1/2 protein is augmented. In addition, the human hedgehog protein-ligand Hh can interact with HHIP proteins, thereby negatively regulating Hh signaling (Wei et al., 2019). And methylation of HHIP has also been found to regulate hedgehog signaling pathways in tumors (Zuo et al., 2018).

The elderly with osteoporosis exhibited lower methylation levels of the HHIP promoter area than the control group, and this corresponded with reduced HHIP expression and increased P1NP and β-CrossLaps, suggesting that HHIP is linked to osteoporosis in the elderly. Related studies have indicated that HHIP has an impact on OC metabolism and controls cell death (Robbins et al., 2012; Li et al., 2016).

### 2.5 Runx2

Runx2 is integral to the transformation of mesenchymal stem cells sourced from bone marrow into OBs, an integral element of bone growth. Related studies have shown that DNA methylation plays a major role in controlling the activities of several genes in OBs and OCs. The methylation of a certain gene has a crucial part in thwarting mesenchymal stem cells from transforming into OBs and OCs, consequently hampering the segregation of these two cell types. The activity and differentiation of OCs influence the development of OBs. Runx2 has been revealed to be an essential element in the growth of OBs, with its influence on the expression of several vital OB characteristics such as osteocalcin, osteochondritis, salivary protein, and type I collagen (Nakashima and de Crombrugghe, 2003; Xu et al., 2015; Xu et al., 2018). Studies to date have indicated that when ovulation is inhibited, it can lead to an increase in methylation of the Runx2 promoter in bone, suppress its transcription and reduce its expression, resulting in a decrease of many OB phenotypic genes and the onset of osteoporosis.

### 2.6 Nrf2

Nrf2 is a transcription factor expressed in a variety of cell types, including OBs, osteocytes and OCs. Nrf2 is thought to be a major regulator of cytoprotective genes against oxidative and chemical injury. Deletion of Nrf2 can lead to pathological alterations in multiple organs. The effect of Nrf2 on bone metabolism was studied using Nrf2 knockout mice under force loading. Nrf2 knockout mice were found to have a 7% (*p* < 0.05) reduction in femoral cancellous bone mineral density, a 40% (*p* < 0.05) reduction in bone formation rate and an 11% (*p* < 0.01) significant reduction in ultimate force compared to wild-type controls of the same age. Force loading experiments showed that the Nrf2 knockout group of mice had lower bone mineralized surface area (RMS/BS, −69%, *p* < 0.01) and lower bone formation rate (rBFR/BS, −84%, *p* < 0.01) than the control group. Furthermore, deletion of Nrf2 resulted in the suppression of antioxidant enzyme and Wnt5a gene expression in primary OBs under force loading experiments. Taken together these results suggest that loss-of-function mutations in Nrf2 in bone impair bone metabolism and reduce bone formation under force loading (Sun et al., 2015).

Professor Kim J-H found that Nrf2 knockout mice exhibited significant bone tissue defects after birth, most severely at 3 weeks of age, when the number of OBs was 12-fold lower than in control animals. Although primary OBs from Nrf2- + mice functioned normally *in vitro*, the colony-forming capacity of bone marrow stromal cells (BMSCs) was significantly lower in these mice than in controls. This suggests that Nrf2 is a key pathway in the regulation of bone metabolism (Kim et al., 2014).

### 2.7 RANK, RANKL, and OPG

RANK and RANKL, as well as the OPG system, are key signaling pathways that regulate bone turnover and are implicated in the development of osteoporosis (Zhao et al., 2020). The RANK gene is an important regulator of the RANK-RANKL-OPG signaling pathway, and Delgado-Calle, Nishikawa et al. investigated the methylation of RANK-RANKL-OPG genes in the bone metabolic pathway. The methylation of genes related to the RANK-RANKL-OPG pathway was investigated. They found that different levels of gene methylation would affect the expression of genes in this pathway and this affects the level of bone turnover, regulates the differentiation and function of OBs and OCs and leads to osteoporosis (Delgado-Calle et al., 2012).

### 2.8 Wnt/β-catenin signaling pathway

Previous research has indicated that there are a variety of ways of dealing with osteoporosis, one of which is the well-known Wnt/β-catenin signaling pathway which is indispensable for the maturation and strengthening of bones (Martínez-Gil et al., 2022). The Wnt/β signaling pathways can be divided into two groups: the canonical pathway that incorporates β-catenin (also known as the Wnt/β-catenin pathway), and the non-canonical pathway that does not contain β-catenin. In recent years, the Wnt/β signaling pathway has been the subject of much research, and is now widely considered to be the most well-understood of the Wnt/β signaling pathways. Mesenchymal stem cells sourced from bone marrow can be effectively transformed into OBs, promoting bone regeneration. As a result, the Wnt/β-catenin signaling pathway has been found to be a crucial factor that decides the destiny of mesenchymal stem cells (Taipaleenmäki et al., 2011). Moreover, the Wnt/β-catenin signaling pathway is a viable option for medical treatment in regards to osteoporosis (Dong et al., 2020).

**FIGURE 1:**
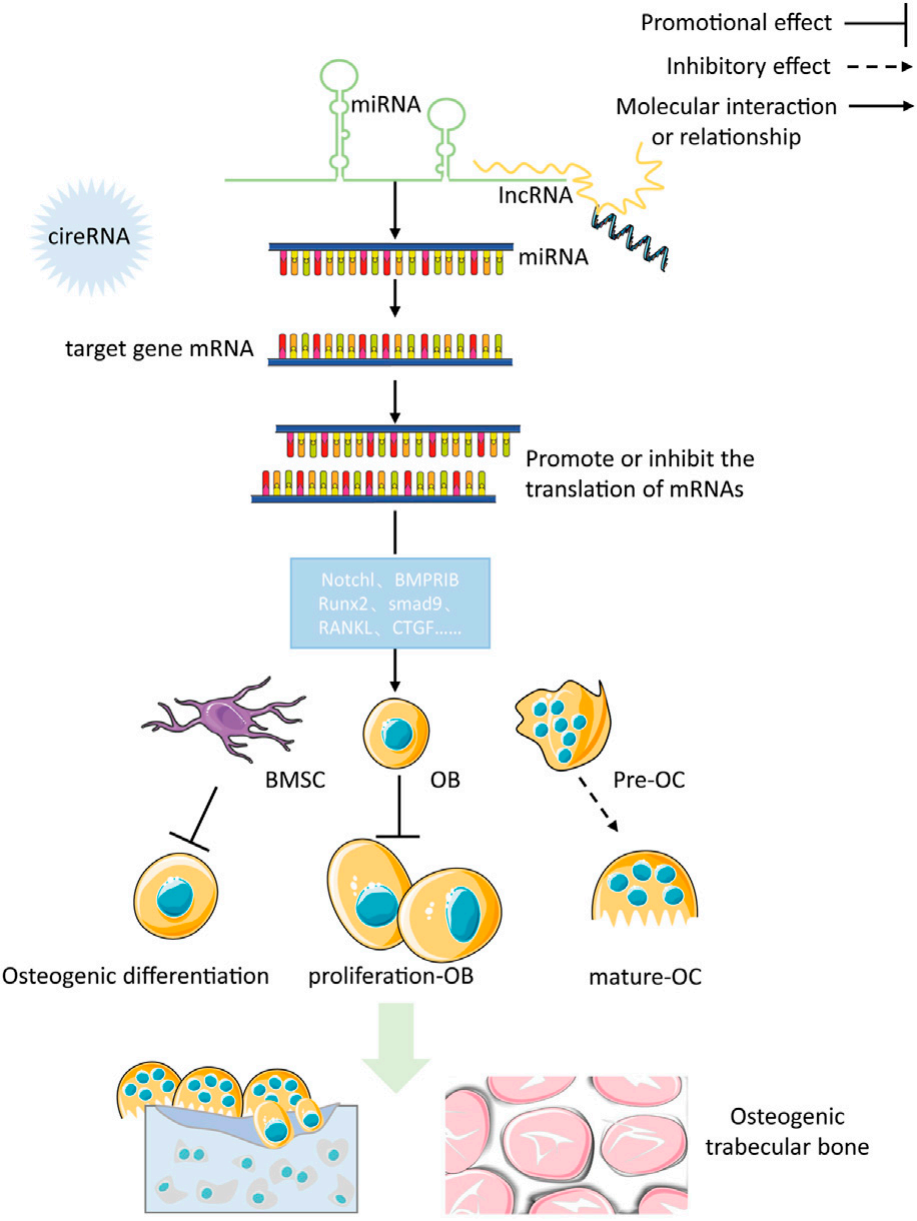
The specific process of Wnt/β-catenin signaling pathway in bone metabolism.

WNTs play a significant role in the growth and maintenance of various organs (including bones) by being secreted as a glycoprotein. The Wnt/β-catenin pathway works in the following manner: Wnt proteins attach to the cell surface receptor, Frizzled (Fz), and the co-receptor, Lipoprotein Receptor-Associated Protein 5/6 (LRP5/6), to activate the Fz receptors and send out signals through the skeleton protein (Dv) and Casein Kinase 1 (CK1). Furthermore, the formation of a complicado incorporating Axin, Colorectal Adenomatous Polyposis Protein (APC) and Glycogen Synthase Kinase 3 (GSK3) triggers intra-cellular signaling pathways. When Wnt signaling is absent, GSK3 adds a phosphate group to beta-catenin, resulting in its destruction through the process of ubiquitination and proteasomal degradation. Without Wnt signaling present, GSK3 modifies beta-catenin, and this is then broken down by the ubiquitin/proteasome system. When Wnt signaling is activated, catenin is gathered in the cytoplasm, migrates to the core and attaches to the TCF/LEF transcription factor, which leads to the activation of genes such as c-myc and cyclinD1, thereby increasing the proliferation of cells or activity. The Wnt/β-catenin pathway signaling is an essential element of the creation of bones, and its disruption can lead to a variety of skeletal and non-skeletal illnesses (Wada et al., 2005). Figure 1 illustrates the specific process of Wnt/β-catenin signaling pathway in bone metabolism.

Shi and his team investigated the mesenchymal stem cells located in the bone marrow of individuals with postmenopausal osteoporosis. The scientists observed that the levels of CTNNB1 gene (catenin β1) - the gene responsible for coding β-catenin - were significantly reduced when compared to healthy control cells. An examination using Western fluorescent blotting demonstrated an augmentation of CTNNB1 mRNA and protein expression levels as bone marrow mesenchymal stem cells differentiated into osteogenic cells (Shi et al., 2020). Patients with osteoporosis are more likely to have type 2 diabetes, which can worsen the condition. The research concluded that type 2 diabetes-induced osteoporosis is mainly a result of weakened bone production rather than accelerated bone breakdown. Type 2 diabetes can cause changes in bone reabsorption and formation, which can result in osteoporosis.

On the other hand, enhancing the aryl hydrocarbon receptor nuclear transporter 1 inhibited GSK-3β in the Wnt/β-catenin pathway, resulting in a remarkable enhancement of BMSCs’ bone regeneration. The catenin pathway has a considerable influence on the regeneration of bone marrow mesenchymal stem cells (Li J. et al., 2017). The diabetes medication alogliptin, which has been utilized in managing type 2 diabetes, may be useful in addressing the issue of osteoporosis. The upregulation of osteogenic markers, for example, recombinant human bone morphogenetic protein two and runt-related transcription factor 2 was observed after the stimulation of the Wnt/β-catenin pathway with keratin, suggesting a significant increase in the alteration of MC3T3-E1 cells in terms of both differentiation and mineralization (Dong et al., 2020).

Exogenous compounds can affect bone development in relation to osteoporosis by altering the Wnt/β-catenin pathway. Yang et al. discovered that FN-1, which is normally present in the extracellular matrix, can have an impact on osteogenesis in individuals suffering from osteoporosis by way of the Wnt/β-catenin signaling pathway. FN-1, and β-catenin interact in a laboratory setting, resulting in the triggering of the Wnt/β-catenin signaling pathway and consequently, the distinguishing of OBs and the formation of bone when FN-1 is expressed. In addition, research suggests that integrin β1 has the ability to bind with FN-1 and is essential for FN-1-induced triggering of the Wnt/β-catenin signaling pathway (Dong et al., 2020). Dickkopf-1 (DKK-1) was demonstrated to prevent alkaline phosphatase activation and calcification caused by AWLNH and PHDL, two peptides sourced from red mussel protein, when administered prior to the proteins. DKK-1 also diminished the mRNA expression of Wnt signaling molecules, implying that AWLNH and PHDL may be able to encourage BMSC differentiation towards OBs across the standard Wnt/β-catenin pathway (Oh et al., 2020).

Changes in some proteins in the Wnt/β-catenin signaling pathway due to osteoporosis can have an effect on bone formation. Researchers have established that biological factors such as genes, medications, and certain metallic elements can influence bone formation by means of the Wnt/β-catenin signaling pathway in a therapeutic setting. Research is anticipated to be key in discovering treatments for orthopedic diseases through further exploration of the Wnt/β-catenin signaling pathway. Research indicates that the excessive stimulation of Wnt/β-catenin signaling could be linked to BMSC aging (Zhang et al., 2011). Despite this, the precise means by which bones are formed remains a mystery. Despite being a major contributor to the formation of bone, the complexity of the Wnt/β-catenin signaling pathway and its many proteins has yet to be fully explored.

### 2.9 Notch signaling pathway

The Notch signaling pathway consists of the following components: Notch receptors, Notch ligands, intracellular effector molecules, and related enzymes (Liu et al., 2010). Notch receptors include Notch1, Notch2, Notch3, and Notch4. It has been shown that Notch 1, 2, and 3 of the Notch receptors are expressed in OBs and are associated with bone metabolism in humans.

The Notch pathway significantly impacts the disease by controlling the activity of both OBs and OCs to maintain equilibrium. Studies have revealed that the Notch signaling pathway plays a part in promoting and hindering the advancement of OBs. In animals lacking Notch1 and Notch2, OB expression is also suppressed, and Notch2 plays a significant role in both. Baldridge’s experiments confirm that deletion of Notch1 and 2 only inhibits the differentiation of OB precursors but has no effect on mature OBs (Baldridge et al., 2010). The Canali’s study confirmed Notch’s inhibitory effect on OB precursors’ differentiation. Notch was also shown to be able to slow down bone breakdown, stop bone deterioration, and protect against osteoporosis at different points in the progression of bone cell growth (Canalis et al., 2013).

The Notch signaling pathway is critical to the proper operation of the human skeletal system. With further research, various experiments have shown that it is involved in controlling the cells that create bone and those that break it down, maintaining cartilage homeostasis, and promoting the growth of tumor cells. The Notch signaling pathway could potentially offer a promising avenue for treating a range of musculoskeletal conditions, as well as a novel target for osteoporosis.

### 2.10 The role of other pathways in osteoporosis

Osteoporosis was linked to the activity of the bone morphogenetic protein (BMP) pathway and the silent mating-type information regulator two homolog 1 (SIRT1) path.

The TGF-β superfamily includes BMP, which acts on cell surface receptors to carry out various tasks (Wu et al., 2016). In contrast, Smad, a transcription factor, is triggered by the BMP receptor, and this is linked to bone growth and development when BMP binds to its receptor. Bone metabolism is regulated through a combination of the classic Smad pathway and the non-standard mitogen-activated protein kinase (MAPK) pathway. Typically, BMPs initially attach to certain receptors on the cellular membrane, resulting in the phosphorylation of BMPR-1 before bonding with Smad proteins (mostly Smad1, Smad5, and Smad8), which then penetrate the nucleus and turn on transcription factors like Runx2 and Osterix to prompt the transformation of BMSCs into OBs (Matsumoto et al., 2012; Lowery and Rosen, 2018). The BMP protein activates the TAK1/2-MEK1/2-ERK1/2 pathway, resulting in the alteration of genetic manifestation of particular genes and the alteration of the non-conventional pathway of osteogenic differentiation. The connection between these two pathways is evident, as SIRT1, a deacetylase, affects multiple pathways, both directly and indirectly. SIRT1 is primarily seen in BMSCs and has an effect on the various pathways of BMSC differentiation. SIRT1 assists in bone regeneration by encouraging the development of OBs and hindering the growth of OCs in bone marrow stem cells. FOXO1 is an O-forkhead transcription factor that functions as an inhibitor of bone formation, is present in OB progenitor cells and reduces Wnt transduction and proliferation in these cells. Conversely, SIRT1 can promote Wnt signaling and bone cell production through its ability to deacetylate FOXO1 and β-catenin (Yao et al., 2018). SIRT1 can also stimulate the development of bone tissue from BMSCs by influencing the Runx2 and TGF-β pathways. SIRT1 additionally controls other transcription factors linked to it. PPARγ2, an essential factor in the development of fat cells, hinders the maturation of BMSCs into OBs either directly through SIRT1 or indirectly by preventing PPARγ antagonists from steering the transformation of BMSCs into fat cells, thereby allowing SIRT1 to guide the formation of BMSCs into OBs. Research on the gene-molecular level showed that miR-146a-5p specifically affects SIRT1 to impede bone cell activity.

## 3 The roles of histone modification on osteoporosis

The term “histone modification” is used to describe the process by which histones are enzymatically modified by methylation, acetylation, phosphorylation, adenylation, and ubiquitination. Protein modification is one of the essential epigenetic modalities regulating OC differentiation (Faulkner et al., 2019). Recent studies have shed light on the critical role of histone modifications in both the development of bone metabolic disorders and OC differentiation (Jing et al., 2018).

Histones are proteins located in the nucleus which have highly similar structures and comprise of five varieties, such as H1, H3, H2A, H2B, and H4. The organization of chromatin, which is affected by histone modifications, can control gene expression and translation, thereby influencing the emergence of related health problems (Wade, 2001; Oton-Gonzalez et al., 2022).

### 3.1 Histone acetylation and OC differentiation

Generally, the amino-terminal lysine of the histone tail is the spot where histone acetylation typically takes place, including H3K9, H3K14, H3K18, H3K23, H4K5, H4K8, H4K12, H4K16, and other related sites. The action of histone acetylases (HATs) leads to histone acetylation, and there are five distinct subfamilies of HATs: HAT1, PCAF/Gcn5, MYST, p300/CBP, and Rtt109 (Marmorstein and Zhou, 2014). Acetyl groups interact with the lysine residues on histones, decreasing their binding with DNA and thus controlling gene expression (Yi et al., 2019).

It has been discovered recently that histone acetylation is a vital factor in the distinction of OCs. The Becn1 promoter region displayed a notable upsurge in H3K9 and H4K8 acetylation levels during OC differentiation as indicated by Raha et al., and this effect was further augmented by the Krueppel-like factor 2 (Klf2) downregulation. Klf2 overexpression, however, had a markedly contrary effect. Klf2 has been identified as a major contributor to the process of adding an acetyl group to a molecule of H3K9 and H4K8 in the Becn1 promoter area (Laha et al., 2019). The study conducted by Vita cˇnik and their team demonstrated that OBs exposed to hypoxia showed an upregulation of HDAC9 and a decrease in lysine acetyltransferase, suggesting that histone acetylation modifications contribute to the control of both osteoclast and osteoblast differentiation (Vrtačnik et al., 2015).

### 3.2 Histone deacetylation and OC differentiation

The acetylation levels of histones are largely ruled by histone deacetylases (HDACs). The human genome contains 18 HDACs, which are used to classify them into four classes located in their enzymatic activity and cellular location. Class I HDACs are HDAC 1-3 and 8, Class II HDACs are HDAC 4-7, 9, and 10, Class III HDACs are Sirtuines 1-7, and Class IV HDAC is HDAC 11 (de Ruijter et al., 2003).

#### 3.2.1 Class I HDAC and OC differentiation

Type I HDACs are mainly situated within the nucleus and possess a unique catalytic structural domain. Kim et al. showed that HDAC1 acts as a repressor in OC differentiation and inhibits OC differentiation through recruitment to the promoters of OC-related genes Nfatc1 and Oscar (Kim et al., 2007). Pham and their team found that deleting the HDAC3 gene had a major impact on the growth of OCs and the production of Nfatc1, Ctsk, and Dc-stamp, which are all linked to OC formation. Research has suggested that the amount of HDAC8 decreases at the beginning of OC differentiation and increases as the process progresses, indicating that it may act in promoting OC differentiation (Jin et al., 2015).

#### 3.2.2 Class II HDAC and OC differentiation

Class II HDAC mainly includes: HDAC4, HDAC5, HDAC6, HDAC7, HDAC9, HDAC10, and in addition to these six. Data suggests that HDAC5 expression is delayed during OC differentiation and its expression level is the highest just before and after OC fusion, a pattern that is similar to that of HDAC4. Knocking down HDAC5 with short hairpin RNA (shRNA) boosts the amounts of OCs, along with a substantial rise in the manifestation of genes associated with OC differentiation and bone breakdown. Wein et al. reported reduced trabecular bone density in HDAC5 knockout mice at 2–3 months of mouse age (Wein et al., 2015). The research conducted by Kim et al. concluded that HDAC7 expression was low at the termination of OB differentiation, and a decrease in HDAC7 was observed to significantly enhance OB differentiation. HDAC7 hinders the production of β-catenin and cyclin D1 when RANKL is present. It appears that HDAC7 has an inhibitory effect on OC differentiation (Jin et al., 2013).

#### 3.2.3 Class III HDAC and class IV HDAC with OC differentiation

Class III HDAC and Class IV HDAC mainly include Sirt1, Sirt2, Sirt3, Sirt4, Sirt5, Sirt6, Sirt7, and HDAC11, and in addition to these eight. A study demonstrated that blocking Sirt1 expression caused a notable rise in the quantity and activity of OCs, along with an increase in FOX protein acetylation, through the RANKL signaling pathway. Jing et al. reported that Sirt2 has a facilitative effect on OC differentiation (Jing et al., 2019). Huh et al. found that Sirt3 knockout mice had significantly increased OC numbers, decreased bone mass, and decreased bone density, suggesting that Sirt3 negatively regulates OC differentiation and function through the AMPK-PGC-1β axis (Huh et al., 2016). Yan et al. demonstrated that Sirt1 expression had notably decreased and miR-506 had significantly increased when bone marrow-derived macrophage (BMM) cells were differentiated into OCs. Bioinformatics prediction and dual-luciferase reporter gene discovery yielded the conclusion that miR-506 had an association with the 3′-UTR of Sirt1, implying that deacetylases could be regulated by microRNA (miRNA) to control OC differentiation (Yan et al., 2019). In summary, different HDACs exert different effects and mechanisms of action on OC differentiation.

### 3.3 Histone deacetylase inhibitors and OC differentiation

Histone deacetylase inhibitors (HDACi) are small molecule compounds with anti-osteoporotic and arthritic effects (in Table 1). Kim et al. showed that class I HDACi MS-275 inhibited OC differentiation and promoted OB formation by suppressing c-Fos protein expression. Animal experiments confirmed the protective effect of MS-275 on IL-1-mediated bone loss in mouse skulls, suggesting that MS-275 exerts anti-osteoporosis effects by regulating the balance of OC and OB functions (Kim et al., 2012). Cantley et al. used microcomputed tomography and histological analysis to examine changes in alveolar bone in olive oil, HDACi MS-275, and 1179.4b treated mice with periodontitis. 1179.4b significantly reduced bone loss, increased bone volume by 3.4%, and was more effective than MS-275 in relation to the control group (Cantley et al., 2011). Guo et al. found that HDACi CI-994 significantly inhibited the distinction between OCs and their effects on bone degradation and downregulated the demonstration of ACP5, CTSK, NFATc1, c-Fos, DC-STAMP, and V-ATPase-d2 genes. Analysis of the phosphorylation of NF-κB subunits IκBα and p65 indicates that CI-994 modulates OC differentiation through NF-κB, which triggers the c-Fos/NFATc1 signaling cascade (Guo et al., 2019).

**TABLE 1:** HADCi and OC differentiation.

Name	Target	Experimental models	Pharmacological action	References
MS-275	HDAC1	OC, OB and Mice	Inhibits OC differentiation and activity, promotes OB formation and inhibits bone loss	Kim et al. (2012)
1179.4b	Class I and II HDAC	Mice (periodontitis)	1179.4b Significant reduction in bone loss and 3.4% increase in bone volume	Cantley et al. (2011)
SAHA	Class I and II HDAC	Mice and Uncle (collagen-induced arthritis)	Reduces joint swelling, bone erosion and bone resorption in mice and rats	Lin et al. (2007)
BML281	Class I and II HDAC	Uncle (collagen-induced arthritis)	Good anti-inflammatory and anti-swelling effect	Lohman et al. (2016)
NW-21	HDAC1 and HDAC2	Uncle (collagen-induced arthritis)	Good anti-osteoarthritic effect	Faulkner et al. (2019)
BML-275	HDAC1	Uncle (collagen-induced arthritis)	Good anti-osteoarthritic effect	Faulkner et al. (2019)
CI-994	HDAC1,2/3	OB	Inhibition of OC differentiation and bone resorption function	Guo et al. (2019)

### 3.4 Histone methylation and OC differentiation

Histone methylation typically occurs at lysine and arginine residues located at the ends of histones such as H3K9, H3K27, H4K20, H3K4, H3K36, H3K79, and H3R17. The modification of H3K4, H3K36, H3K79, and H3R17 *via* methylation is strongly correlated to the initiation of gene expression (Greer and Shi, 2012; Farooq et al., 2016).

There is a growing amount of research to suggest that histone methylation, which is carried out by three subgroups of transferases, SET, PRMT, and DOT1, engages in the control of OC differentiation (Völkel and Angrand, 2007). Das et al. found that the histone methylation regulator PTIP maintains bone marrow integrity and normal hematopoietic function by promoting OC differentiation. Das et al. found that the histone methylation regulator PTIP maintains bone marrow integrity and normal hematopoietic function by promoting OC differentiation (Das et al., 2018). Gao et al. reported that H3K79 methyltransferase DOT1L is an essential regulator of histone methylation. During OC differentiation, DOT1L expression and H3K79 methylation levels were significantly upregulated. Silencing or inhibiting DOT1L activity significantly promoted OC differentiation and increased OC surface area and bone loss levels in de-ovalized mice, suggesting that DOT1L is a new target for mediated OC differentiation (Gao and Ge, 2018). Kim and colleagues noted that H3K27me1 is essential for the H3 N-terminal protein to be broken down through MMP-9 (Kim et al., 2018). Fang and colleagues suggested that the histone methyltransferase EZH2 facilitates OC differentiation by decreasing the levels of IRF8, a factor that blocks OC formation (Fang et al., 2016).

### 3.5 Histone demethylation and OC differentiation

Histone demethylation is a modification in which demethylases demethylate histones, including the KDM and JMJD families. Recent studies have suggested that histone demethylation is a key factor in regulating skeletal issues, particularly when it comes to OCs differentiation (in Table 2) (Chen et al., 2015). A study demonstrated that Kdm3C knockout mice increased alveolar bone destruction in mice induced by experimental periodontitis or pulp exposure. BMS isolated from Kdm3C knockout mice had increased OC formation and Kdm3C loss. It is suggested that Pg-LPS promotes p65 phosphorylation and nuclear translocation in exposed cells. Kdm3C promotes OC differentiation through NF-κB signaling (Lee et al., 2019). Liu et al. found that Jmjd7 expression were significantly decreased during OC differentiation, and silencing of Jmjd7 expression promoted the expression and bone resorption activity of OC-related genes c-fos, Dc-stamp, Cts K, Acp5, and Nfatc1, suggesting that Jmjd7 is associated with OC differentiation and regulate osteolytic activity (Liu et al., 2018).

**TABLE 2:** Histone demethylase and OC differentiation.

Demethylase	Function	Mechanism	References
KDM3C	Promoting OC differentiation	Regulation of OC differentiation through the NF-kB signaling pathway	Lee et al. (2019)
JMJD7	Inhibition of OC differentiation	Encouraging the manifestation of OC-associated genes c-Fos, Dc-stamp, CtsK, Acp5, and NFATc1	Liu et al. (2018)
JMJD3	Inhibition of OC differentiation	Silencing of the Jmjd3 gene reduced the demethylation of NFATc1 and H3K27me3	Yasui et al. (2011)
JMJD5	Inhibition of OC differentiation	Promoting the degradation of NFATc1 protein negatively regulates OC differentiation	Youn et al. (2012)
KDM4B	Promoting OC differentiation	Inhibition of KDM4B activity activates KDM1A and inhibits the release of precursor inflammatory factors	Kirkpatrick et al. (2018)
NO66	Promoting OC differentiation	NO66-overexpression causes massive bone loss and stimulates the articulation of OC-related genes Dickkopf1, Cathepsin K, and NF-kB in mice	Sinha et al. (2019)

### 3.6 Other histone modifications and OC differentiation

Other histone modifications have been studied relatively little in OC differentiation. Obri et al. demonstrated that PTH promotes OC differentiation by promoting RANKL expression by regulating HDAC4 ubiquitination by Smurf2, a regulator of ubiquitination (Obri et al., 2014).

### 3.7 Relationship between histone modifications and OB

Kaneki et al. found that TNF significantly upregulated the articulation of Smurf1 and Smurf2 in OB precursors and primary OBs and increased the degradation of endogenous Runx2 protein, an OB-specific transcription factor, suggesting that histone ubiquitination is indispensable for bone metabolic diseases (Kaneki et al., 2006).

Histone methylation can occur at lysine and arginine residues, with the addition of one, two, or three methyl groups in the presence of methyltransferases. Amino acid methylation at different sites has different properties; methylation of H3K4, H3H36, and H3K79 loosens the chromatin state and is transcriptionally active, whereas methylation of H3K9 and H3K27 makes chromatin more condensed and transcriptionally silent (Marini et al., 2016). In addition, high levels of methylation are more significantly altered in function. Histone methyltransferases can be classified into SET and non-SET structural domains based on the sequence of the catalytic structural domain.

It has been shown that SETD2 mediates the enrichment of its target gene, H3K36me3, a lipopolysaccharide-binding protein, maintains its chromatin in an active state, actively engages in transcription initiation and elongation, and regulates the conversion of BMSCs to OBs *in vitro* and *in vivo* (Wang et al., 2018a). This implies that SETD2 and its downstream target genes may be effective targets for metabolic bone diseases. The EZH2 gene encodes a lysine methyltransferase and inhibition of EZH2 activity was found to control the hypermethylation level of H3K27me3, which in turn stimulated OB maturation, and was verified using the inhibitor GSK126 (Galvan et al., 2021). The key to EZH2 inhibition of osteogenesis is the disruption of BMP2 signaling and the combination of EZH2 inhibitor and BMP2 treatment stimulates osteogenic differentiation in BMSCs while reducing the high cost and adverse effects of BMP2 alone (Dudakovic et al., 2020). The synergistic application of epigenetic modulation and osteosynthetic drugs can therefore be used as a therapeutic strategy to improve osteogenesis.

## 4 The roles of miRNA, lncRNA and circRNA in the development of osteoporosis

Over the past several years, lncRNAs have been the topic of much scientific inquiry. RNA strands with a length greater than 200 nucleotides generally do not result in a significant amount of protein production. Many assume lncRNAs are simply a consequence of transcription and are not biologically relevant. A recent study has uncovered that lncRNAs are intricately connected to physiological processes and are linked to the emergence and progression of human illnesses. LncRNA can impact target genes through various mechanisms, including transcription, post-transcription, and epigenetic regulation, and can be involved with the emergence and advancement of illnesses like osteoporosis (Rajpathak et al., 2014; Terashima et al., 2017).

NcRNAs are typically split into two sections: housekeeping and regulatory. Regulatory ncRNAs are essential for epigenetic control and can be broken down further into miRNAs, PIWI-interacting RNAs, siRNAs, lncRNAs, enhancer RNAs, and promoter-associated RNAs, based on their size and purpose. Among them, miRNA is the first of the ncRNAs. LncRNAs and circRNAs are additional major classes of miRNAs.

**FIGURE 2:**
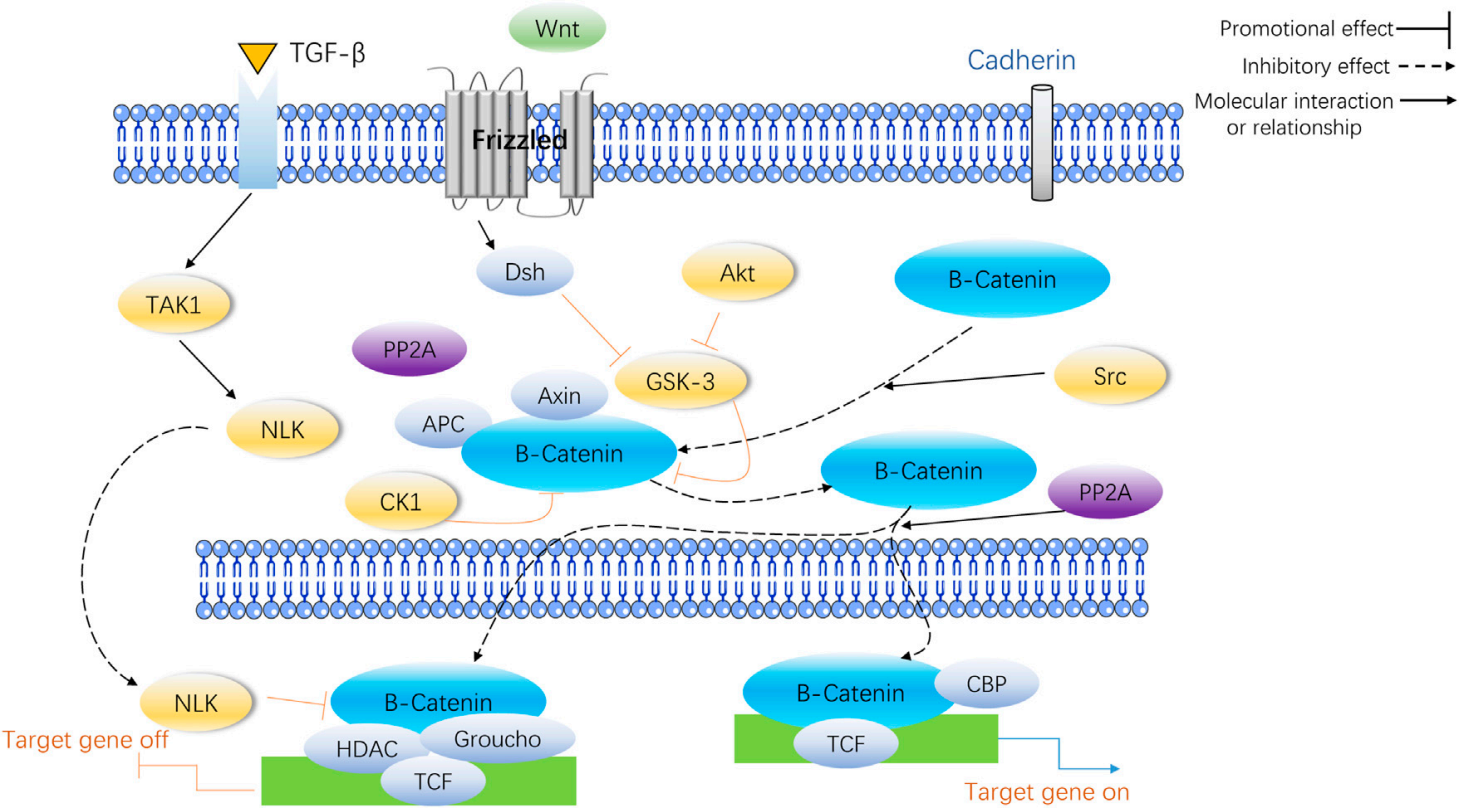
miRNA, lncRNA and circRNA affect bone metabolism and participate in the occurrence and development of osteoporosis.

Changes to gene expression can occur through epigenetic modifications, without any changes to the genetic code. A mounting amount of data has revealed that miRNAs, lncRNAs, and circRNAs are integral to the control of bone growth and breakdown as scientific study progresses. One relative research indicated that postmenopausal osteoporosis patients showed a different level of expression for 13 miRNAs, 70 lncRNAs, and 260 circRNAs compared to healthy individuals (Jin et al., 2018). Data presented in Figure 2 reveals that miRNAs, lncRNAs, and circRNAs have an impact on bone metabolism and are involved in the progress of osteoporosis.

### 4.1 miRNA-mediated regulation of OB and OC differentiation

Small non-coding RNAs, known as miRNAs, are made up of roughly 22 nucleotides and they can control up to half of the genetic information within a genome. MiRNAs form a complex with the 3′-UTR of mRNAs by binding to it, which leads to either halting protein production or causing the mRNA to be degraded. This activity encourages the post-transcriptional control of gene expression. Research has revealed a strong correlation between miRNAs and bone metabolism, and a disruption of their expression has been linked to an impairment of bone metabolism and osteoporosis (Chang et al., 2020). Consequently, miRNA gene therapy shows great promise as a treatment for osteoporosis.

Numerous investigations have illustrated that miRNAs are able to induce the distinguishing of OB through controlling molecules that are involved in its differentiation, thereby leading to successful bone reconstruction and preventing osteoporosis from progressing. iR-291a-3p increased the life of BMSCs, their capacity to form bones, and their ALP activity by focusing on DKK1. Transfection of siRNA-291a-3p mimics caused a rise in vocalization of the osteogenic genes Runx2, DMP1, and ALP, activated the Wnt/β-catenin pathway, and augmented glucocorticoid-induced osteogenic differentiation of BMSCs into osteoporosis (Chang et al., 2020). Research has demonstrated that miR-96 has the ability to influence SOST, resulting in the activation of the Wnt/β-catenin pathway and consequently boosting osteogenic differentiation. The expression of miR-96 caused an elevation in ALP activity, mineralized nodules, and OB viability (Ma et al., 2019a). MiR-216a contributes to bone metabolism by modulating the c-cbl-mediated PI3K/AKT pathway, thus safeguarding osteogenesis and stimulating osteoblastic distinguishing between various tissues and creating skeletal structures (Pan et al., 2019). In contrast to the effects of certain miRNA promoters, certain miRNAs, when expressed in high amounts, hindered OB differentiation and quickened osteoporosis progression. The findings suggested that miR-125b hinders OB formation by targeting NKIRAS2 and Runx2, suppresses osteogenic differentiation of BMSCs through BMPR1b, and concurrently activates NF-κB signaling. In addition, the *in vivo* tests of bone repair transplantation revealed that bone marrow mesenchymal stem cells with decreased miR-125b expression exhibited an extraordinary capability of repairing bone flaws (Li X. et al., 2017). Fan et al. found that when miR-203a-3p was suppressed, it caused smad9 to prevent OB differentiation and stimulated the Wnt/β-catenin pathway (Fan et al., 2019). To conclude, miRNA has the capacity to direct the specialization of BMSCs by manipulating genes and signaling pathways engaged in the activity of bone formation, making it an attractive candidate for determining the cause and managing osteoporosis. Moreover, knowledge of miRNA expression regulations will help create treatments that are specifically aimed at osteoporosis (in Table 3).

**TABLE 3:** miRNAs play a role in controlling the biochemical processes of bone development.

Regulation of OB and OC	miRNAs	Target genes	Signaling pathways
Promoting OB differentiation	miR-291a-3p Chang et al. (2020)	DKK1	Wnt/β-catenin
	miR-2861 Li et al. (2009)	BMP2	-
	miR-96 Ma et al. (2019a)	SOST	Wnt/β-catenin
	miR-216a Pan et al. (2019)	c-Cbl	PI3K/AKT
	miR-199a-3P Lu et al. (2021)	Erk2	-
	miR-150-3p Qiu et al. (2021)	Runx2	-
	miR-1275 Zou et al. (2021)	Runx2	-
	miR-106 Arumugam et al. (2018)	Runx2	-
Inhibition of OB differentiation	miR-125b Li X. et al. (2017)	BMPR1b	-
	miR-103-3p Sun et al. (2021)	METTL4	
	miR-30a Kim et al. (2021)	Runx2	
	miR-23a Kim et al. (2021)	Runx2	
	miR-203a-3p Fan et al. (2019)	Smad9	
Promoting OC differentiation	miR-21 Madhyastha et al. (2019)	RANKL, PTEN	PI3K/AKT
	miR-223 Li J. et al. (2017)	Runx2	-
	miR-19a Li J. et al. (2017)	Runx2	-
	miR-214 Sun et al. (2018)	PTEN	PI3K/AKT
	miR-31 Sun et al. (2018)	RANKL	-
Inhibition of OC differentiation	miR-17 Sun et al. (2018)	RANKL	-
	miR-20a Shi et al. (2014)	RANKL	-
	miR-503 Xie et al. (2019)	RANK	-

Studies have shown that different miRNAs can affect Rankel-induced OC differentiation. First, during OC generation, RANKL upregulates miR-21 and activates the PTEN-directed PI3K/AKT pathway to advance OC generation and bone resorption (Madhyastha et al., 2019). The manifestation of Runx2 is controlled by miR-223 and miR-19a, which then modify the RANK/RANKL pathway, thereby influencing the development of bone destruction caused by osteolysis (Li J. et al., 2017). Finally, as a crucial controller of bone development, miR-214 advances OC activity by aiming at PTEN through the PI3K/AKT pathway and mediates OC-OB crosstalk (Sun et al., 2018). On the contrary, the miRNAs specified beneath hindered OC development. In this research, miR-31 was observed to impede the formation of OCs induced by RANKL, and miR-17 and miR-20a were fashioned to decrease the amount of RANKL present in OCs and obstruct OC differentiation (Mizoguchi et al., 2013; Shi et al., 2014). MiR-503 suppresses RANK, thus blocking RANK from connecting to RANKL and suppressing RANKL-promoted OC differentiation and activation (Xie et al., 2019). The above studies suggest that as miRNAs are intensively studied in the life sciences, more and more miRNAs have been shown to mediate the developmental process of osteoporosis by affecting the differentiation, activation, and activity of OC, and these specific miRNAs may become new targets for targeted drug therapy for osteoporosis. Several miRNAs involved in the control of OB and OC are displayed in Table 3.

### 4.2 lncRNA-mediated regulation of OB and OC differentiation

RNAs longer than 200 nucleotides that are not involved in protein synthesis, commonly known as lncRNAs, are pivotal in providing essential functions such as epigenetic, transcriptional, and post-transcriptional regulation. Furthermore, lncRNAs can serve as a buffer for miRNA, modify mRNA expression, and be involved in activities such as inflammation and cell metabolism.

LncRNAs can impact the process of bone formation through altering the genetic components that control bone metabolism. First, lncRNA AK023948 regulates phosphorylation levels of AKT in OBs from estrogen-deficient osteoporosis rats to activate the PI3K/AKT pathway, thereby controlling the spread of OB (Wang et al., 2020). Second, the lncRNA MIR22HG downregulates PTEN and activates PTEN/AKT signaling to advance the transformation of bone marrow stem cells into bone cells (Jin et al., 2020). In addition, it was ascertained that in lncRNA CRNDE knockout mice, OB proliferation and differentiation were reduced and resulted in low bone mass. When its expression is elevated, it regulates OB proliferation and differentiation by initiating the Wnt/β-catenin pathway (Mulati et al., 2020). Contrary to the regulatory effects of the above lncRNAs, the overexpression of lncRNA DANCR inhibited Runx2 transcription and osteogenic differentiation and resulted in the inactivation of p38 MAPK that mediates BMSC proliferation and osteogenic differentiation (Zhang et al., 2018).

In comparison, osteoporosis patient BMSCs had considerably higher amounts of HOTAIR lncRNA than healthy individuals, and suppressing its expression could particularly enhance ALP activity and the quantity of calcified nodules. Studies conducted elsewhere revealed that HOTAIR lncRNA blocked the production of Wnt/β-catenin pathway-related proteins and prevented BMSCs from undergoing osteogenic differentiation (Shen et al., 2019). In addition, lncRNAs that inhibit OB differentiation *via* the Wnt/β-catenin pathway include lncRNA DANCR, lncRNA p21, and lncRNA AK045490 (Yang et al., 2019a; Li et al., 2019). Despite the indication that lncRNAs engage in the control of bone metabolism, further exploration is needed to understand the development and management of osteoporosis. Recent advancements in technology have further illuminated the ways in which lncRNAs control bone metabolism. It is expected that controlling the development of bone marrow mesenchymal stem cells and strengthening their ability to form bone will be advantageous for bone tissue engineering.

One related research has demonstrated that lncRNAs can control OC production by influencing the manifestation of certain objective mRNAs. TUG1 lncRNA, for instance, has the ability to control OC growth and PTEN-induced cell death (Han et al., 2019). A further investigation established that NFATc1 is a principal transcription factor in OC differentiation and that AK077216, a long ncRNA, was markedly augmented during OC formation, which, in turn, increased the expression of NFATc1 (Liu et al., 2019). Lee and colleagues discovered that lncRNA-jak3 could trigger OC differentiation by increasing NFATc1 expression, which confirms that lncRNA-jak3 is critical for OC differentiation *via* the jak3/NFATc1/Ctsk pathway (Lee et al., 2019). The presence of lncRNAs was found to impede OC differentiation, and these lncRNAs showed an inverse relationship with the intensity of osteoporosis. The activity of the related genes that were activated by RANKL was inhibited by lncRNA BMNCR, thus halting the process of OC differentiation (Chen et al., 2019a). In comparison, lncRNA GAS5 encourages OC cell death by decreasing the amount of miR-21 present (Cong et al., 2020). Another study found that IL-6 can promote OC differentiation and activation. LncRNA-NEF has the ability to suppress IL-6 production, thus affecting osteoporosis. Higher lncRNA-NEF levels are associated with shorter surgical procedures and lower recurrence rates after treatment (Ma et al., 2019b). Ultimately, these lncRNAs which are influencing OC development could be a potential game changer in the treatment of osteoporosis. However, studies on the effects of lncRNAs on OC still need to be completed. Unraveling the precise ways in which lncRNAs control bone metabolism could offer promising clinical prospects for the treatment of osteoporosis**.** Some lncRNAs involved in the control of OB and OC are shown in Table 4.

**TABLE 4:** lncRNAs are involved in controlling the process of bone formation and breakdown.

Regulation of OB and OC	lncRNAs	Target genes	Signaling pathways
Promoting OB differentiation	lncRNA AK023948 Wang et al. (2020)	-	PI3K/AKT
	lncRNA GAS5 Li et al. (2020)	Smad7mRNA	UPF1/Smad7
	lncRNA MIR22HG Jin et al. (2020)	PTEN	PTEN/AKT
	lncRNA CRNDE Mulati et al. (2020)	-	Wnt/β-catenin
	lncRNA-MALAT1 Zang et al. (2022)	miRNA-143	-
	lncRNA-MSC-AS1 Zhang et al. (2019b)	miRNA-140e-5p	miRNA-140e-5p/BMP2/Smad
Inhibition of OB differentiation	lncRNA DANCR Zhang et al. (2018)	Runx2/p38/MAP	Wnt/β-catenin
	lncRNA-ANCR Peng et al. (2018)	miRNA-758	Notch2
	lncRNA HOTAIR Shen et al. (2019)	miR-17-5p	Wnt/β-catenin
	lncRNA p21 Li et al. (2019)	-	Wnt/β-catenin
	lncRNA AK045490 Yang et al. (2019a)	-	Wnt/β-catenin
	lncRNA-MEG3 Wang et al. (2017)	miRNA-133a-3	miRNA-23b/RUNX 2
	lncRNA-TUG1 Teng et al. (2021)	miRNA-23b	
	lncRNA CCAT1 Hu et al. (2021)	miR-34a-5P	
	lncRNA AK039312 Yin et al. (2021)	miR-199B-5P	
	lncRNA AK079370 Yin et al. (2021)	miR-199B-5P	
Promoting OC differentiation	lncRNA TUG1 Han et al. (2019)	PTEN	
	lncRNA-SNHG1 Yu et al. (2021)	miRNA-181c-5p	SFRP1/Wnt/β-catenin
	LINC00311 Wang et al. (2018b)	-	Notch
	lncRNA FTX Liu et al. (2019)	miR-137	-
	lncRNA AK077216 Lee et al. (2019)	NFATc1	-
	lncRNA MIRG Ling et al. (2019)	miR-1897	miR-1897/NFATc1
	lncRNA CRNDE Li et al. (2018)	-	PI3K/Akt
Inhibition of OC differentiation	lncRNA BMNCR Chen et al. (2019a)	RANKL	-
	lncRNA GAS5 Cong et al. (2020)	miR-21	-
	lncRNA-NEF Ma et al. (2019b)	IL-6	-

### 4.3 circRNA-mediated regulation of OB and OC differentiation

CircRNA is a particular kind of RNA molecule that has a consistent presence and is difficult to break down. Its main feature is a closed loop with the 3′ and 5′ ends reverse spliced and covalently bonded, which can effectively resist the effects of RNA exonucleases. CircRNAs have numerous miRNA-binding sites that enable them to act as miRNA-absorbing sponges, thus involving regulation. Consequently, circRNAs could control PO by connecting with miRNAs.

The regulation of circRNA in OB differentiation is a current research hotspot. Some circRNAs associated with the control of OB and OC are shown in Table 5. Research indicates that mmu_circ_003795 expression increases when MC3T3-E1 cells undergo osteogenic differentiation, which then leads to higher levels of COL15A1 and osteopontin due to its competition with miR-1249-5p (Wu et al., 2020). Furthermore, after BMP2 induction in MC3T3-E1 cells, ALP levels and activities were increased, and Runx2 levels were upregulated. Conversely, circRNA_0016624 activates miR-98, increases BMP2 expression, and promotes OB differentiation (Yu and Liu, 2019). A separate investigation determined that PIK3R1 expression stimulated the growth of MC3T3-E1 cells and prevented cell death. The researchers found that circRNA AFF4 was a promoter of MC3T3-E1 cell proliferation, which is important for bone healing, as its expression was raised and it acted to inhibit miR-7223-5p and thereby increase PIK3R1 levels during fracture recovery (Mi et al., 2019). In contrast to the above mechanism, the expression levels between circRNA IGSF11 and the expression of miR-199b-5p had an inverse relationship, and this correlation was upregulated during osteogenesis in BMSCs. IGSF11 circRNA knockdown increases miR-199b-5p expression and promotes OB differentiation (Zhang et al., 2019a). To sum up, circRNAs have a critical influence on OB development, growth, and death and may become potential treatments for osteoporosis and fractures. However, as shown in Table 5, the effects of the circular RNAs mentioned above on OB differentiation have been established. Compared to miRNAs and lncRNAs, circRNAs have been less reported in the relevant literature, so an in-depth study of their relationship with osteoporosis will be a future topic.

**TABLE 5:** circRNAs are implicated in the control of bone metabolism.

Regulation of OB and OC	circRNAs	Target genes
Promoting OB differentiation	mmu_circ_003795 Wu et al. (2020)	miR-1249-5p
	circRNA_0016624 Yu and Liu (2019)	miR-98
	circRNA AFF4 Mi et al. (2019)	miR-7 223-5p
	circ_0048211 Qiao et al. (2020)	miRNA-93-5p
	circ-VANGL1 Yang et al. (2019b)	miRNA-217
	circRUNX2 Yin et al. (2018)	miR-203
	circ_0011269 Xu et al. (2020)	miR-122
	circ_0026827 Ji et al. (2020)	miR-188-3p
	circFOXP1 Shen et al. (2020)	miR-33a-5p
	circ_0006393 Wang et al. (2019)	miR-145-5p
	circ_0006215 Ji et al. (2021)	miR-942-5p
	circRNA-circHmbox1 Liu et al. (2020)	miRNA-1247-5P
	circRNA-0076906 Wen et al. (2020)	miR-1305
Inhibition of OB differentiation	circRNA IGSF11 Zhang et al. (2019a)	miR-199b-5p
	circ_0006859 Zhi et al. (2021)	miR-431-5p
	circ_0001275 Xu et al. (2021)	miR-377
	circ_0003865 Wang et al. (2021)	miR-3653-3p
Promoting OC differentiation	circRNA_28313 Chen et al. (2019b)	miR-195a
	circRNA_005108 Chang et al. (2020)	miR-31
	circRNA_009934 Miao et al. (2020)	miR-5107
	circHmbox1 Ramasamy et al. (2014)	miR-1247-5p
Inhibition of OC differentiation	circRNA_0021739 Guan et al. (2021)	miR-502-5p
	circ_0001795 Liu et al. (2021)	miR-378

Relevant studies on the positive regulation of OC by circRNAs are relatively few, with circRNA_28313 and circRNA_005108 being more clearly studied. During RANKL + CSF1-induced OC differentiation, circRNA_28313 was significantly upregulated and competitively bound to miR-195a to raise the amount of CSF1 expressed and thus promote OC differentiation (Chen et al., 2019b). CircRNA_005108 encourages the formation of actin rings in OBs by binding miR-31 to increase the expression of RhoA (Chang et al., 2020). The studies on the negative regulation of OC by circRNA were even more minimal. It was found that circRNA_0021739 overexpression reduced miR-502-5p levels and inhibited OC differentiation (Guan et al., 2021). By examining the gene expression levels between individuals with osteoporosis and those without it, Jin et al. discovered that circRNA_0021739, circRNA_0011269, circRNA_0019693, circRNA_0005245, and circRNA_0010349 were most significantly downregulated during OC production. Thus, further validation of their regulatory role on OC will be a hot spot for future research (Jin et al., 2018).

## 5 Conclusion and perspective

Epigenetic processes are essential in managing the activity of OBs and OCs, which is responsible for regulating the production and destruction of bone in reaction to external cues, such as the communication between muscles and bones and between bones and the brain. Research has indicated that elements in the environment like diet, age, stress, and exercise may alter epigenetics by altering methylation of osteogenic transcription factors, modulating muscle-derived miRNAs and histones, and thereby influencing gene expression and metabolism in bones.

The existence of epigenetic mechanisms provides potential molecular markers and identifying compounds that can be used to avert and address osteoporosis, in turn suggesting that treatment can be initiated by altering epigenetic modifications. Based on DNA methylation, genes, proteins, or pathways such as SOST, Erα, DMNT, HHIP, Runx2, Nrf2, RANK, RANKL, OPG, Wnt/β-catenin signaling pathway, and Notch signaling pathway provide important directions for clinical breakthroughs in osteoporosis. Histone modifications also provide important targets for the treatment of osteoporosis from an epigenetic perspective. Studies have shown that miRNAs are significant therapeutic objectives for osteoporosis, for example, epimedium-Xenical medicine regulating miRNA-144 for secondary osteoporosis. Meanwhile, a series of epigenetically active drugs discovered in recent years may also play a role in treating osteoporosis, such as the BET protein antagonist JQ1, which reduces bone loss in OVX rats (Baud’huin et al., 2017). NMP regulates OB/OC balance and inhibits inflammatory cytokines in the treatment of osteoporosis in OVX rats (Gjoksi et al., 2015). In terms of diagnosis, a range of epistatic modifications, led by circulating miRNAs, are seen as emerging biomarkers of bone disease and are engaged in the early diagnosis and identification of bone disease (Heilmeier et al., 2016). LncRNA and circRNA are also important breakthrough directions.

Although there has been extensive research conducted into the genetics and epigenetics of osteoporosis, the intricate biological processes behind it remain largely enigmatic. Genomics that are used for practical applications and other histological investigations including epigenetics in osteoporosis have severe restrictions, such as difficulties in obtaining large numbers of human bone tissue samples, poor cohort characteristics, or lack of healthy controls. Current trials are not yet sufficient to explain the pathological changes in humans. The next step should be to look for similarities and differences in epigenetic mechanisms within and outside the human body and to establish links between *in vivo* and *in vitro* studies. Going forward, we should integrate the genetic and epigenetic molecular markers identified in previous studies to build a new risk assessment model covering the dynamic interaction of genetics-environment- ageing, and complement it with precise individual intervention programmes to reduce the recurrence rate and ultimately achieve early screening and efficient prevention of osteoporosis.
